# Online multimodal rehabilitation programme to improve symptoms and quality of life for adults diagnosed with long COVID-19: a Randomised Clinical Trial protocol

**DOI:** 10.3389/fpubh.2023.1222888

**Published:** 2023-09-07

**Authors:** Sandra León-Herrera, Rosa Magallón-Botaya, Bárbara Oliván-Blázquez, Lucía Sagarra-Romero, Carlos Martín Jaurrieta, Fátima Méndez-López

**Affiliations:** ^1^Institute for Health Research Aragón (IIS Aragón), Zaragoza, Spain; ^2^Department of Psychology and Sociology, University of Zaragoza, Zaragoza, Spain; ^3^Department of Medicine, Faculty of Medicine, University of Zaragoza, Zaragoza, Spain; ^4^GAIAS Research Group, Department of Health Sciences, Faculty of Health Sciences, Zaragoza, Spain

**Keywords:** long COVID-19, telerehabilitation, multimodal, multidisciplinary, quality of life, effectiveness

## Abstract

**Background:**

Long COVID is a multisystemic condition which affects quality of life and implies a multidisciplinary treatment approach. There is still limited evidence on management techniques for this syndrome. “Telerehabilitation” could be an important tool when addressing the symptoms of this patients with the aim of increasing their quality of life. The purpose of this trial is to analyse the effectiveness of an online multimodal rehabilitation programme to improve the symptomatology of people with long COVID and their quality of life.

**Methods:**

A pragmatic randomised controlled trial will be performed with two parallel groups: (1) usual treatment by the primary care practitioner (Treatment as usual, TAU; control group) and (2) TAU plus the use of an online multimodal rehabilitation programme, including videoconferences and content published on a Moodle platform (intervention group). The data will be collected before and after the intervention. A follow-up will take place 3 months later.

**Discussion:**

There is still a lack of knowledge regarding the management of the symptoms of long COVID. This creates the need to add scientific evidence about the care of this disease, considering that multidisciplinary social and health teams can offer the necessary care so that these patients can recover their previous quality of life.

**Clinical trial registration**: The protocol for this study was registered with the ISRCTN Registry [registration number: ISRCTN15414370] on 28 December 2022.

## 1. Introduction

In March 2020, when the coronavirus disease 2019 (COVID-19) pandemic was declared, hardly anyone would have thought that this disease would affect the physical and mental health of the entire world in the way that it did, and that it could even be considered a chronic condition (1, 2) As the pandemic has progressed, we have seen that after the initial acute infection by severe acute respiratory syndrome coronavirus 2 (SARS-CoV-2), some people develop a long-lasting multi-organ syndrome with various symptoms (general, respiratory, neurological, cardiovascular, gastrointestinal and psychosocial symptoms), a phenomenon that also occurs with many other viral disorders. The World Health Organization (WHO) called this condition “long COVID” (3–5). In the long term, these symptoms lead to a significant decline in the functional status and quality of life of people who experience them. Indeed, these symptoms limit the ability of people to perform basic activities of daily living (ADL), such as bathing or dressing, as well as occupational, social and leisure activities (6–8).

People with long COVID exhibit heterogenous symptoms and this multisystemic condition implies the need for a multidisciplinary treatment approach (9, 10). In current clinical practice, the treatment is based on managing the symptoms and the affected systems of each patient (11). In other words, a single care strategy is not possible for all people with long COVID; it is necessary to develop personalised care programmes (12).

Several guidelines about the management of long COVID have already been published (13–17). However, these clinical guidelines are not fully supported by scientific evidence, leading to a large practice gap (17). This lack of knowledge about the management and treatment of symptoms by general practitioners (GPs) has led to people with long COVID feeling that their concerns are not being taken seriously (18). Consequently, these patients have expressed their willingness to try anything (over-the-counter medications, different types of therapy, home remedies, dietary changes, supplements, etc.) to ameliorate the disabling symptoms. These types of measures can be harmful if they are not supervised by a qualified medical professional, a fact that supports the urgent need to generate evidence concerning the clinical management of long COVID (19, 20). However, this also shows that in the research carried out about long COVID, it is crucial to listen to the narratives and experiences of patients, turning them into co-producers of scientific knowledge (21).

There have been significant technological advances in recent years (22). The use of technology has been raised as an alternative to provide various rehabilitation services remotely (23). This approach increased especially during the COVID-19 confinement, when face-to-face contact between patients and health care professionals was limited, and the population was highly virtualized (24, 25). This type of care is commonly called ‘telerehabilitation’, a promising means that includes different techniques, among which are messages or phone calls, videoconferences or the use of Internet platforms (26). These types of programmes make it possible to provide, in real or deferred time, safe and supervised care at the patient’s home or other important environments (27). The advantages of telerehabilitation include greater participation and commitment on the part of the patients, a more rigorous follow-up of the intervention, personalisation of the treatment plans and, above all, a good perception on the part of those who receive it, because it favours accessibility and reduced waiting times (28).

Although more studies are needed to guarantee an adequate level of scientific evidence, telerehabilitation could represent an important element in the comprehensive rehabilitation of patients in the acute phase and with sequelae of COVID-19 (10). Aiyegbusi et al. (29) already indicated that videoconferences could be an effective tool in the follow-up of people with long COVID and that, as long as it is feasible, digital therapy programmes could be implemented through a platform that includes non-pharmacological interventions, such as respiratory rehabilitation. Moreover, Samper-Pardo et al. (30) are studying the use of mobile applications to improve the quality of life of people with long COVID, and Ozduran et al. (31) are analysing the effectiveness of YouTube videos to manage pain, a very significant symptom in this patient population.

This trial will investigate long COVID and its management by multidisciplinary social health care teams. The objective is to analyse the effectiveness of a multimodal rehabilitation programme, with online and in-person components, to ameliorate the characteristic symptoms of long COVID and, consequently, to improve quality of life. A secondary objective is to analyse the factors associated with the effectiveness of this intervention in people with long COVID.

## 2. Methods

### 2.1. Research design and study registration

**Figure 1:**
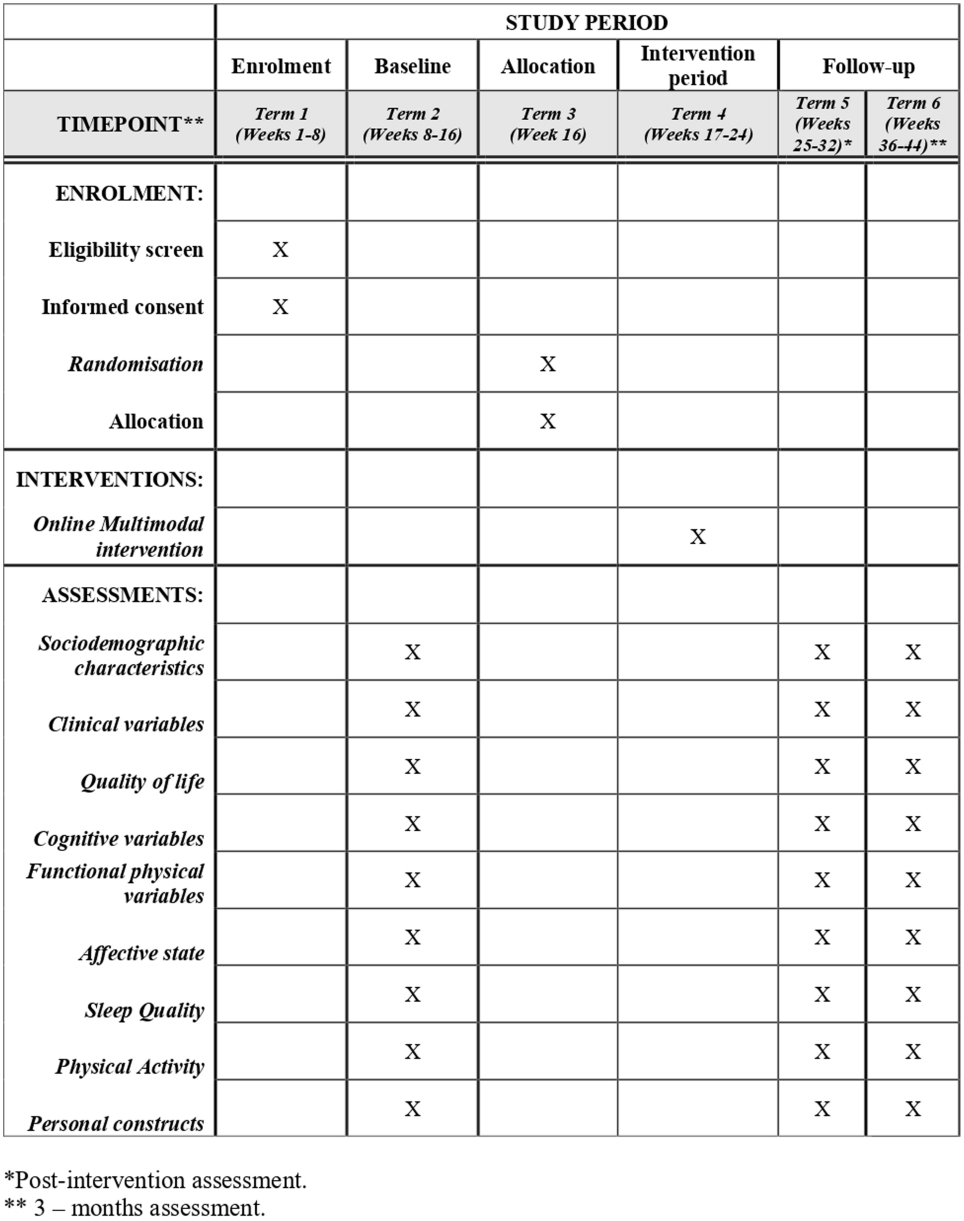
SPIRIT schedule of enrolment, allocation, intervention and assessments.

This pragmatic randomized controlled trial will include two parallel groups: (1) usual treatment by the primary care practitioner (Treatment as usual, TAU; control group) and (2) TAU plus the use of an online multimodal rehabilitation programme (intervention group). The results will be reported by following a pre-established plan based on the CONSORT guidelines (32) to compare the two groups. Figure 1 shows a systematic overview of the study.

The protocol for this study was registered with the ISRCTN Registry (registration number: ISRCTN15414370) on 28 December 2022, and is being reported using the SPIRIT (Standard Protocol Elements: Recommendations for Interventional Trials) (Supplementary materials).

### 2.2. Patient inclusion and sample size

The study population will follow the following inclusion criteria: (1) people over 18 and under 80 years of age; (2) with persistent COVID symptoms for at least 3 months since acute infection, and are not explained by an alternative diagnosis; (3) who are part of long COVID associations in Spain. The exclusion criteria will be the presence of a serious uncontrolled medical illness that may interfere with adherence to the rehabilitation programme; receiving structured rehabilitation or psychotherapeutic treatment by health care professionals; participation in another clinical trial in the last 6 months; considerable risk of suicide; pregnancy or lactation; and the presence of any medical, psychological or social problems that could seriously affect the patient’s participation in the study.

The research team will inform the presidents of the different Spanish long COVID associations about this project; they will then pass the information to the members of these organisations. Those interested in participating should contact the project researchers by using the contact details provided in the invitation letter sent to the associations. The evaluating researcher will then contact the participant and determine whether the patient can be included in the study (based on the inclusion and exclusion criteria). Recruitment and baseline assessments will be carried out until the sample size has been reached.

In accordance with the results obtained in the literature (33), to detect a mean difference of 20 points on the physical scale of the 36-Item Short Form Health Survey Questionnaire (SF-36), with a standard deviation (SD) between the groups of 36.16, an alpha value of 0.05 and a power of 80%, 53 subjects per group will need to be recruited (106 subjects in total). We will increase this value by 10% to cover potential losses in the study, so the minimum sample required will be 116 subjects. Considering a mental scale, to detect a mean difference of 20 points, with an SD of 29.99 between the groups, an alpha value of 0.05 and a power of 80%, 35 subjects per group will need to be recruited (70 subjects in total). This number will be increased by 10% to counter possible losses, so the sample size is 77 subjects. Based on these calculations, the minimum sample required for the study will be 58 subjects per group (116 subjects in total). The sample size has been calculated using the Fisterra Guide for Determination of sample size (34). The formulae used have been added as Supplementary materials.

At the time of writing 90 participants (77.5% of the target sample size) have been recruited.

### 2.3. Randomisation, allocation and masking of the study groups

Patients will be randomised once baseline data have been collected. Individual randomisation will be performed by an independent statistician using a computer-generated random number sequence with no restrictions. The sequence will be concealed throughout the study. Given the nature of the interventions, the patients will not be blind to their allocation. The information for the random allocation sequence will be implemented by phone by a research assistant (RA), who will state the type of treatment assigned for each new patient. The participants in the intervention group will be informed about what the intervention is and where and when they should carry it out. In addition, the RA will request that participants do not inform other researchers of their group allocation.

### 2.4. Data collection and monitoring

A RA will collect the data, and another will enter and encode the identification data. All RAs handling the data will be blind to patient allocation, as will the RA performing outcome assessment and data analysis. All the information collected will be treated in accordance with the provisions of the current legislation on the protection of personal data.

Any serious unexpected adverse event or result will be discussed by the trial management committee (the authors of this protocol). There are no plans to discontinue or modify the interventions, to improve adherence or to promote participant retention. The committee will monitor enrolment, treatment, attrition rates and any concerns related to the study. The reasons for abandonment will also be recorded.

### 2.5. Intervention

Patients assigned to the control group will follow the treatment as usual provided by their GP.

Patients assigned to the intervention group will follow the treatment as usual provided by their GP and will participate in a multidisciplinary online multimodal rehabilitation programme. The programme will be aimed at addressing the symptoms of people with long COVID and improving their quality of life. This objective will be pursued by providing exercises and therapeutic recommendations related to physical activity, respiratory rehabilitation, cognitive rehabilitation, diet, sleep hygiene, use of community resources and emotional management.

#### 2.5.1. Intervention tools

The rehabilitation will be carried out through two techniques that are typically used in telerehabilitation programmes. On the one hand, a group videoconference will be convened weekly, in which follow-up and personalisation of the contents of the intervention will be carried out. On the other hand, a Moodle (Modular Object-Oriented Dynamic Learning Environment) Platform will be used as support; it will contain all of the content provided to patients during the videoconferences.

The programme will consist of eight videoconference sessions (one per week) of approximately 1.5 h each through the Google Meet application. Three groups will be planned for each session so that patients can choose which group to attend according to their availability, and with the goal of achieving the maximum possible assistance. In addition, dividing all patients into three smaller intervention groups (a maximum of 18 people per group) will make the intervention more individualised.

The sessions will be oriented towards managing each type of symptomatology (cognitive, respiratory, physical, etc.) while addressing the different impacts on quality of life (work, social and emotional impact) and offering recommendations that can positively influence the health of this type of population (Table 1). The same content will be provided in all three groups. The sessions will not be recorded. If a person misses a session, he/she will be able to make up for it during the schedule of one of the other two groups.

**Table 1:** Content of the videoconference sessions.

Videoconference sessions	
Session 1	Management of neurological and neurocognitive symptoms Cognitive stimulation guidelines
Session 2	Management of respiratory symptoms
Session 3	Management of physical symptoms
Session 4	Diet recommendations to address long COVID symptoms
Session 5	Sleep disorders Sleep hygiene
Session 6	Emotional impact of long COVID
Session 7	Social impact of long COVID
Session 8	Compilation of therapeutic recommendations Doubts and questions

Also, a Moodle Platform will be created, where all the material and content provided throughout the telerehabilitation programme will be available. The platform will be divided into eight sections, whose name and content will coincide with each videoconference session (Table 1). For example, in the first section, patients will be able to find content related to the neurocognitive symptoms of long COVID and how to deal with them. This same subject will be discussed during the first videoconference of the programme.

Patients will be able to find the following content on the Moodle Platform:

- Access links to videoconferences (Google Meet application);
- PowerPoint presentations displayed during videoconferences;
- Lists of exercises and therapeutic recommendations, including cognitive exercise books, respiratory physiotherapy videos, list of diet recommendations, etc.;
- Forums to debate and interact on aspects addressed during the videoconference;
- Other resources of interest (news, glossary of terms, web pages or social networks of interest, etc.).

Instructions for how to access the weekly videoconferences and the online platform will be provided to each patient before starting the intervention, both verbally (by phone) and in writing (by email).

To carry out the rehabilitation programme, patients may use any of their personal devices: mobile phone, computer, tablet, etc., although the health team will recommend using a computer in order to avoid visual fatigue, especially when performing cognitive exercises.

In the event that any of the patients have difficulties accessing videoconferences or the platform, the rehabilitation team will try to solve them as soon as possible together with the patient.

#### 2.5.2. Content of the online multimodal rehabilitation programme

The content of each of programme sessions will be based on scientific evidence that aims to address the symptoms of long COVID and, therefore, to improve quality of life of this patient population (13–17). The patients must adapt this intervention to their state of health, choosing the recommendations and advice that correspond to their symptoms and their specific health problems derived from this disease. Those recommendations or exercises that may cause some harm or discomfort to the participants must be discontinued immediately.

The multimodal rehabilitation sessions will consist of the following content:

##### 2.5.2.1. Approach to neurological and neurocognitive symptoms

Neurological symptoms are very prevalent in people with long COVID. These may include tiredness, sleep disorders, movement disorders, paraesthesia and cognitive deficits (35). The latter have been defined with the term “brain fog’”, which is characterised by feeling slow, confused and disoriented; having difficulties staying focused; presenting some forgetfulness; and the inability to find the correct word (36, 37). Currently, no specific treatment has been approved for this cognitive impairment in people with long COVID, although it has been determined that cognitive stimulation may be useful in some cases (38).

The content of this session will include: a description of the types of neurological symptoms typical of long COVID; treatment of neurocognitive symptoms; the benefits of performing cognitive stimulation exercises; personalised recommendations about what cognitive exercises can be practiced, and how and when to do it; and implementation of these treatments.

##### 2.5.2.2. Approach to respiratory symptoms

Long COVID often includes respiratory symptoms. The most common are usually shortness of breath, coughing and chest pain (39). Physiotherapy programmes focused on improving respiratory function can be useful in managing these persistent symptoms (40).

The content of this session will include: a description of the types of respiratory symptoms of long COVID; treatment of respiratory symptoms; the benefits of performing breathing exercises; personalised recommendations about what breathing exercises can be practiced, and how and when to do it; and implementation of these treatments.

Patients will be advised to use a pulse oximeter during these exercises. Whenever the patients experience any anomaly in the pulse oximeter values (resting oxygen saturation < 88%) (41) as well as any sensation of pain or pressure in the chest, they should automatically stop performing these exercises and consult what happened with the health professional during the sessions so that he/she can personalize the exercises to that specific person.

##### 2.5.2.3. Approach to physical symptoms

People with long COVID describe drastically reduced physical function. Hence, it becomes difficult or impossible for them to perform some ADL (42). Research on physical activity and long COVID is still insufficient, although there is scientific evidence for benefits in other pathologies with similar characteristics. Existing recommendations on physical activity and long COVID emphasise the importance of personalised care based on the symptoms that each person presents (43, 44).

The content of this session will include: a description of the types of physical symptoms of long COVID; treatment of physical symptoms; the benefits of physical activity; personalised recommendations about what activity and physical exercises can be practiced, and how and when to do it; and implementation of these treatments.

As with the breathing exercises, patients will be recommended to use a pulse oximeter while performing physical exercises, as well as to stop performing the exercises and consult a professional when noticing any abnormality in the values or any type of pain.

##### 2.5.2.4. Recommendations for a healthy diet

The Clinical Guide for Long COVID patient care of the Spanish Society of General and Family Physicians (SEMG) recommends considering food and diet in the management of this disease to fill possible nutritional deficiencies (16). Some nutrients such as folic acid, vitamin D, omega-3 fatty acids and vitamin B12, present in the Mediterranean diet, are involved in the proper functioning of metabolism and the immune system, so they could be useful in the recovery process of these patients (45, 46).

The content of this session will include: recommendations for a healthy diet based mainly on adherence to the classic Mediterranean diet.

##### 2.5.2.5. Approach to sleep and rest disorders

Sleep disorders (decreased sleep quality, daytime sleepiness, insomnia, sleep apnoea, nightmares, etc.) are common among the persistent symptoms of long COVID (47).

The content of this session will include: a description of the types of sleep disorders present in long COVID; information about different treatments for sleep disorders; a sleep hygiene programme; and recommendations for sleep and rest.

##### 2.5.2.6. Managing emotional impact

There is extensive research on the emotional impact on people with persistent symptoms of COVID-19. Anxiety, depression, post-traumatic stress disorder, mood fluctuations and sleep disorders are some of the consequences that long COVID can have on the mental health of people who have the disease (48).

The content of this session will include: a description of the emotional impact of long COVID; treatments aimed at addressing the emotional impact; recommendations about emotional management; an explanation of the first steps to take to meditate; and implementation of two meditation dynamics.

##### 2.5.2.7. Behavioural activation and promotion of participation in the community

The limiting persistent symptoms of long COVID, the consequences on mental health and the reduction in quality of life have a great impact on the organisation of the lives of people with this disease. They are limited in their ability to perform basic ADL, as well as their occupational, social and leisure activities in the community. This isolation is increased by feelings such as fear or uncertainty, barriers to accessing the health system or stigmatisation (7, 8, 49).

The content of this session will include: a description and examples of community health assets (HA); the benefits of using resources offered by the community itself; and personalised recommendations about what community resources can be used, and how and when to do it.

##### 2.5.2.8. Compilation of important aspects, resolution of doubts, and farewell

The last session, held the last week of the programme, will aim at collecting the most important therapeutic recommendations and at resolving doubts and questions that have arisen and that have not already been resolved in the previous sessions.

It is important to highlight that although each session is mainly aimed at addressing a specific type of symptomatology or impact on quality of life, the exercises described in the previous sessions will continue to be carried out and doubts that arise will be resolved. For example, in session 1, some cognitive stimulation guidelines will be explained, and cognitive stimulation exercises will be provided to patients to perform at home. In session 2, the patients will be able to express problems that have arisen when carrying out these exercises at home, and the therapist will even be able to carry out some other exercises to evaluate the progress of the patients with these symptoms.

### 2.6. Outcomes and measures

Data will be collected from questionnaires completed by the patients at the beginning and the end of the intervention. The assessors who administer the questionnaires will call and make an appointment with the patients following a list of first and last names arranged alphabetically and will not know the type of treatment given to each patient. Once the intervention is finished, an RA will contact the study participants and make an appointment to complete the questionnaires again. Hence, the effect that the intervention has had on the symptoms and quality of life will be evaluated. A follow-up will also be carried out after 3 months. The instruments that will be used are listed in Table 2. All assessments will last between 30 and 45 min per patient. If any of the patients experience cognitive fatigue and feel unwell due to the long duration of the assessments, they may be stopped and continued another day.

**Table 2:** Study variables and measurement instruments.

Instrument	Assessment area
Gender, age, marital status, education, household, occupation	Sociodemographic
Contraction of COVID-19, number of residual symptoms and their severity	Clinical variables
36-Item Short Form Health Survey Questionnaire (SF-36) (50, 51)	Quality of life
Montreal Cognitive Assessment (MoCA) (52–54) Memory Impairment Screen (MIS) (55, 56) Semantic Verbal Fluency Test (Animals) (57, 58) Stroop Colour and Word Test (59)	Cognitive variables
Sit to stand test 30 s (60)	Functional physical variables
Hospital Anxiety and Depression Scale (HADS) (61)	Affective state
Insomnia Severity Index (ISI) (62)	Sleep quality
International Physical Activity Questionnaire (IPAQ-SF) (63)	Physical activity
Self-efficacy scale (64) Health Literacy Europe Questionnaire (HLS-EUQ16) (65) Patient Activation Measure Questionnaire (PAM) (66)	Personal constructs
Likert scale	Satisfaction with the program

#### 2.6.1. Primary outcome

The main variable will be quality of life, evaluated through the SF-36 (67). This questionnaire measures eight dimensions of health: physical function, physical role, aches and pains, general health, vitality, social function, emotional role and mental health; it also includes a declared health evolution item. The eight dimensions define two main components of health: a physical summary component and a mental summary component. In them, scores above or below 50 indicate, respectively, a better or worse state of health than the mean of the reference population. Items are scored on a Likert-type scale from 1 to 3.5 or 6, depending on the type of item. The total score of the eight scales ranges from 0 to 100, with higher scores indicating better health. The validated Spanish version of the questionnaire will be used (68).

#### 2.6.2. Secondary outcomes

##### 2.6.2.1. Sociodemographic variables

Age, gender, marital status, education, household and occupation will be collected.

##### 2.6.2.2. Clinical variables

The date of contracting COVID-19 and the number and severity of persistent symptoms measured by Visual Analog Scale (69) will be collected. Persistent symptoms include: low-grade fever (37–38°C), fever (> 38°C), chills without fever, headaches, dizziness, tiredness or fatigue, sore throat, cough, dyspnoea, loss of smell and/or taste, blurred vision and other eye problems (dry eyes, increased dioptres, conjunctivitis), bruising, hair loss, tachycardia, myalgia, joint pain, orthostatic hypotension, chest pain, back pain (cervical, thoracic and/or lumbar), gastrointestinal symptoms, urinary symptoms (overactive bladder, infections), neurological symptoms (spasms, tingling, etc.), cognitive impairment (memory loss, reduced attention, mental fog), altered menstrual cycle, loss of libido or erectile dysfunction, and other symptoms that the participants consider persistent (70–72).

##### 2.6.2.3. Cognitive variables

The official Spanish version of the Montreal Cognitive Assessment (MoCA) (52–54) will be used to assess the presence of cognitive impairment in the participants of this study. This is a test with adequate internal consistency (Cronbach’s alpha = 0.76) and assesses six cognitive domains: memory, visuospatial capacity, executive function, attention/concentration/working memory, language and orientation. Of the total score of 30 points, a correction of one point can be made in the case of subjects with less than 12 years of schooling. The cut-off point of this scale for the detection of mild cognitive impairment in its original version is 26. This test has been used in previous research to assess cognitive impairment in people with long COVID (73, 74).

The presence of short-term memory impairment will be measured by using the Spanish version of the Memory Impairment Screen (MIS). This short test assesses the presence of memory disturbances by testing free recall (without clues) or selective recall (with semantic clues) of four words. Dementia screening has adequate interobserver (0.85) and test–retest (0.81) reliability. One point is awarded per word recalled with the help of semantic clues, and two points per word obtained by free recall. The total score ranges from 0 to 8, with a score of ≤4 points indicating possible deterioration (55, 56).

The Semantic Verbal Fluency Test (Animals) will be used to assess whether verbal fluency is affected. The test–retest reliability is 0.68. This test consists of counting the number of correct words within the ‘Animals’ category, reproduced for 1 min. Normally, a healthy person will be able to reproduce about 16 words per minute (57, 58).

The Stroop Colour and Word Test will be used to assess processing speed. This test consists of three phases spread over three different pages. On the first page, the words “red”, “green” and “blue” are printed in black ink, randomly repeated in columns. On the second page, the element “XXXX” appears repeatedly and in columns, printed in red, green or blue ink. On the third page, the words “red”, “green” and “blue” are printed in red, green or blue, but the words do not match the colour in which they are printed. The patient must, by columns, read words or name the ink colours as quickly as possible for 45 s (75, 76). There are scales corrected for age (7–80 years) to calculate the total score for this test. In the various existing versions, this test has shown consistent reliability, obtaining indices of 0.85, 0.81 and 0.69 for the three direct scores (59).

##### 2.6.2.4. Functional physical variables

The Sit to Stand Test will be used to measure the strength and endurance of the lower limbs (60). The 30-s version of this test will be used; it is specifically designed to detect respiratory diseases (58). It evaluates endurance at high power or speed in terms of muscular endurance or strength by recording the number of times a person can stand up and sit down completely in 30 s. This instrument had good test–retest reliability (0.84 < R < 0.92). The 30-s Sit to Stand Test has been translated into Spanish and used in patients with COVID-19 (77).

##### 2.6.2.5. Affective variables

The affective state will be evaluated by using the Hospital Anxiety and Depression Scale (HADS) questionnaire (61). This is a self-report-based scale designed to screen for depression and anxiety disorders in primary care settings. It consists of 14 items that assess symptoms of anxiety and depression (HADS-A and HADS-D, respectively), each item corresponding to a 4-point scale (0–3), with a score range from 0 to 21 for anxiety symptoms and depression symptoms. Higher scores indicate more severe symptoms. To facilitate its use in international trials, this test has been translated into multiple languages, including Spanish (78).

##### 2.6.2.6. Sleep quality

The Insomnia Severity Index (ISI) will be used to measure the participants’ sleep quality (60). This is a self-report that measures the patient’s perception of nocturnal and daytime symptoms of insomnia: difficulty falling asleep or staying asleep, waking up early in the morning, satisfaction with the current sleep pattern, interference with the daily functioning, perceived impairment attributed to sleep deprivation and level of distress or worry caused by the lack. This scale has seven items, each of which is scored from 0 to 4. The total score ranges from 0 to 28, with a higher score indicating more severe insomnia. The Spanish version of the ISI (79) has shown adequate internal consistency (Cronbach’s alpha = 0.82). This test has already been used in other studies about long COVID (80).

##### 2.6.2.7. Personal constructs

Self-efficacy will be measured by using the Self-Efficacy Scale-12 (64). The original scale consists of 17 items that are scored using a 5-point Likert scale. Woodruff and Cashman (81) derived a factor structure representing the three aspects underlying the scale: willingness to initiate the behaviour (“Initiative”), willingness to strive to complete the behaviour (“Effort”) and persistence in the face of adversity (“Persistence”). These are the three factors that this scale evaluates. Five items have been excluded due to low item-remainder correlations and ambiguous wording, resulting in a 12-item version (GSES-12). The total scale has a Cronbach’s alpha of 0.69, and its internal consistency is 0.64 for “Initiative”, 0.63 for “Effort” and 0.64 for “Persistence” (82).

The Health Literacy Europe Questionnaire (HLS-EUQ16) will be used to measure the health literacy of the participants (79). This is defined as the motivation of the population, their knowledge and their individual capacity to understand and make decisions regarding the promotion and maintenance of their health. The questionnaire consists of 16 items, each scored from 1 (very easy) to 4 (very difficult). The total score is obtained by adding the scores of all the items. The final score can be converted into a dichotomous answer: very difficult and difficult = 0; easy and very easy = 1. Poorer health literacy is indicated by higher scores. The official Spanish version presents high consistency (Cronbach’s alpha = 0.982) (83).

The activation of the patient about his own health will be measured by using the Patient Activation Measure (PAM) (66). This tool evaluates the skills, knowledge and confidence perceived by the patient when carrying out health self-management activities. It comprises 13 items that are evaluated on a Likert-type scale from 1 (totally disagree) to 4 (totally agree). The resulting score ranges from 13 to 52, with higher scores indicating higher levels of activation. There is only one official version in Spanish that applies to patients with chronic disease. It has a reliability of 0.98 and a parametric discrimination index of 6.64 (84).

##### 2.6.2.8. Personal satisfaction with the content and tools

In the last assessment, using a Likert-type scale from 0 to 10, the level of satisfaction both with the content of the program and with the tools used for its development will be assessed.

### 2.7. Statistical analysis

First, a descriptive analysis (frequencies and percentages for categorical variables; and means and standard deviation for continuous variables) and a univariate analysis [one-way analysis of variance (ANOVA) for quantitative variables and the chi-square test for qualitative variables] will be used to examine the data and to test whether there are baseline differences between the groups after randomisation. Second, to answer the main objective—whether there are differences between treatment groups regarding their effectiveness in improving quality of life, linear mixed-effects models (LMEMs) (85) will be used. Cohen’s *d* will be calculated from the estimated mean values of SF-36 and its standard deviations (SD) at baseline (86). Moreover, LMEMs with the same aforementioned components will be used to analyse whether there are differences between the groups with respect to the improvement of the secondary variables.

The results from the trial will be presented as regression coefficients for predicting change in primary and secondary outcomes with 95% confidence intervals. LMEMs will be tested against a Bonferroni-adjusted alpha level of 0.01 (0.05/5) (87).

Data collection and statistical analysis will be performed by using Excel software, R statistical software (version 3.6.2) (88) and SPSS software (version 25.0) (89).

## 3. Discussion

The number of people with long COVID, most of them young and without previous pathologies, continues to increase. However, due to the limited scientific evidence, this pathology is still largely unknown in primary care, and patients still find it difficult to receive recognition, support, medical evaluation and treatment for this condition (90, 91).

There is no standard protocol for the treatment of long COVID. Programmes are being designed with the aim of improving the physical health and mental well-being of these patients; however, some key points such as the content of these programmes, the method of offering them and their duration, among other factors, are still being investigated. These programmes include pharmacological treatments and other approaches such as pulmonary and cardiac rehabilitation, fatigue treatment, etc. These types of disease management are primarily directed at symptom control (92).

Long COVID is considered a multisystem syndrome, and > 100 different symptoms—general, physical, respiratory, cognitive, psychological, etc.—have been reported. Hence, the treatment approach requires an interdisciplinary vision that includes, among others, physical rehabilitation, cognitive rehabilitation, respiratory physiotherapy and psychological intervention, and whose objective is to recover the functional state prior to the disease. In addition, due to the heterogeneity of each case, rehabilitation programmes must be personalised (1, 93).

In recent years, especially during the COVID-19 pandemic when face-to-face contact was limited, the use of digital technology has optimised traditional medical care, improving safety, efficacy and adherence to treatments (94).

The evidence supports the use of digital interventions, highlighting their potential in the control of persistent physical and psychological symptoms of COVID-19, and improving the quality of life of people with these symptoms (9, 28, 95–98). However, Rinn et al., in their scoping review, state that research is still needed on individualized digital interventions that better suit the requirements of these patients (99).

To the best of our knowledge, this is the first randomised controlled trial that will be carried out with the objective of analysing the effectiveness of an interdisciplinary and online multimodal intervention, through videoconferences, in addressing the symptoms of people with Long COVID and in improving their quality of life.

This project has strengths and limitations. Among the strengths are that its national and remote character through videoconferences will offer people with long COVID the opportunity to expand their support network and to learn about similar experiences and resources offered in other communities. The online platform used for support will give the patients access to all of the content, and the videoconferences will promote adherence to the programme, as well as the personalisation of the therapeutic recommendations offered in it. Among the limitations of the study are possible technological barriers as well as the lack of knowledge and skills related to information and communication technologies by some users. Another limitation may be the possible dropout of study participants, which might happen due to reinfections that cause their health status to worsen, or to the search for other face-to-face, individualised and more personalised health care resources during the study. Nonetheless, the reasons for dropout and other problems that may arise throughout the research project will be collected by the researchers in accordance with the Mechanisms of Action in Group-based Intervention (MAGI) framework (100).

## 4. Ethics and dissemination

Ethical approval was granted by the Clinical Research Ethics Committee of Aragon (PI22/482). The procedures carried out for the production of this work were adjusted to the ethical standards of the aforementioned committee and with the 1975 Declaration of Helsinki.

Study participants will receive written information about the conditions of the study, voluntary participation, the right to leave the study at any time, data security, and the publication of anonymized results. In addition, all subjects will sign a written informed consent form; their data will be anonymised and used only for research purposes. The study participants will be informed of the results. The ethics committee will be notified of any modification or change in the protocol.

The trial results will be submitted for publication in peer-reviewed journals. In addition, the study and its results will be disseminated through conferences conferences and researchcongresses.

## 5. Conclusion

There is still a great lack of knowledge regarding the management of long COVID and its myriad symptoms. Hence, there is an urgent need to continue increasing the scientific evidence and to delve into this disease, considering that the multidisciplinary socio-sanitary teams can offer the necessary care so that these patients achieve their recovery.

## Ethics statement

Ethical approval was granted by the Clinical Research Ethics Committee of Aragon (PI22/482). Written informed consent to participate in this study will be obtained from the patients/participants prior to their participation.

## Author contributions

SL-H, RM-B, BO-B, and FM-L designed the study protocol with important contributions of LS-R and CMJ. SL-H and BO-B wrote the main manuscript text. All authors contributed to the article and approved the submitted version.

## Funding

This work is supported by Carlos III Health Institute, grant number PI21/01356, FEDER Funds “Another way to make Europe.” The funders have no role in study design, data collection, analysis, decision to publish or manuscript preparation. The funding organisation will conduct an audit trial once a year.

## Conflict of interest

The authors declare that the research was conducted in the absence of any commercial or financial relationships that could be construed as a potential conflict of interest.

## Publisher’s note

All claims expressed in this article are solely those of the authors and do not necessarily represent those of their affiliated organizations, or those of the publisher, the editors and the reviewers. Any product that may be evaluated in this article, or claim that may be made by its manufacturer, is not guaranteed or endorsed by the publisher.
