# Supplementary material for: Online multimodal rehabilitation programme to improve symptoms and quality of life for adults diagnosed with long COVID-19: a Randomised Clinical Trial protocol
Source: Front Public Health. 2023 Sep 7;11:1222888. doi: 10.3389/fpubh.2023.1222888 (PMC10513419; doi:10.3389/fpubh.2023.1222888)
Supplement: Supplementary file 1 [file Data_Sheet_1.PDF]

GOBIERNO DE ARAGON
Departamento de Sanidad

Informe Dictamen Favorable
Trabajos académicos

C.I. PI22/482

30 de noviembre de 2022

Dña. María González Hinjos, Secretaria del CEIC Aragón (CEICA)

CERTIFICA

1º. Que el CEIC Aragón (CEICA) en su reunión del día 30/11/2022, Acta Nº 21/2022 ha evaluado la propuesta del Trabajo:

Título: Intervención psicoeducativa para la mejora de la calidad de vida y la sintomatología de personas con COVID persistente: Ensayo Clínico Aleatorizado

Doctoranda: Sandra León Herrera
Directoras: Bárbara Oliván Blazquez y Rosa Magallón Botaya

Versión protocolo: Versión 3 (28/11/2022)
Versión documento de información y consentimiento: Versión 3 (28/11/2022)

2º. Considera que

- El proyecto se plantea siguiendo los requisitos de la Ley 14/2007, de 3 de julio, de Investigación Biomédica y los principios éticos aplicables.
- El Tutor/Director garantiza la confidencialidad de la información, la obtención de los consentimientos informados y el adecuado tratamiento de los datos, en cumplimiento de la legislación vigente y la correcta utilización de los recursos materiales necesarios para su realización.

3º. Por lo que este CEIC emite **DICTAMEN FAVORABLE a la realización del proyecto**.

Lo que firmo en Zaragoza

GONZALEZ HINJOS MARIA - DNI 03857456B

Firmado digitalmente por GONZALEZ HINJOS MARIA - DNI 03857456B
Fecha: 2022.12.01 15:10:27 +01'00'

María González Hinjos
Secretaria del CEIC Aragón (CEICA)

Tel. 976 71 5836 Fax. 976 71 55 54 Correo electrónico mgonzalezh.ceic@aragon.es
