# Supplementary material for: Online multimodal rehabilitation programme to improve symptoms and quality of life for adults diagnosed with long COVID-19: a Randomised Clinical Trial protocol
Source: Front Public Health. 2023 Sep 7;11:1222888. doi: 10.3389/fpubh.2023.1222888 (PMC10513419; doi:10.3389/fpubh.2023.1222888)
Supplement: Supplementary file 2 [file Data_Sheet_2.DOCX]

Supplementary Material

**Effectiveness of an Online Multimodal Rehabilitation Programme to improve symptoms and quality of life for people diagnosed with Long COVID-19: a Randomised Clinical Trial Protocol.**

**Sandra León-Herrera^1,2^, Rosa Magallón-Botaya^1,3^; Bárbara Oliván Blázquez^1,2*^; Lucia Sagarra-Romero^4^; Carlos Martín Jaurrieta^1^; Fátima Méndez-López^1^**

*** Correspondence:** Bárbara Oliván Blázquez: bolivan@unizar.es

**
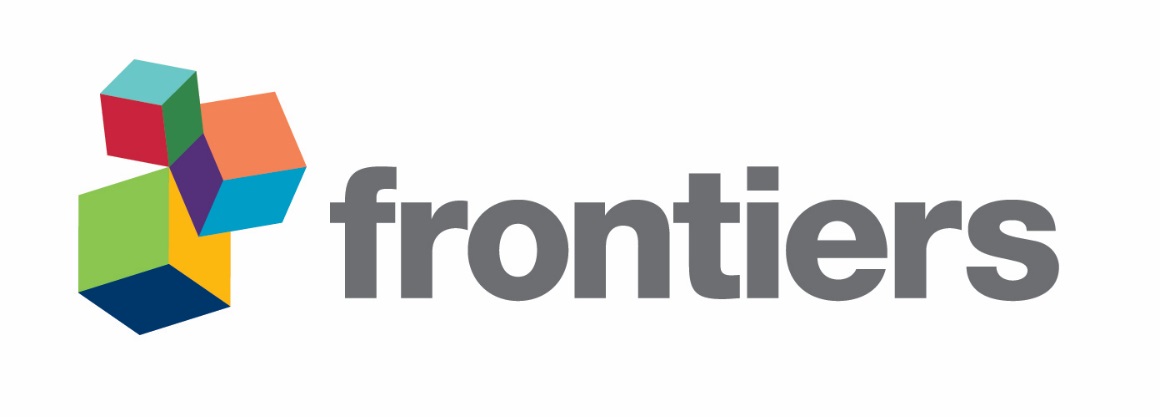
**

| $n=\frac{{2\left( Z\alpha+ Z\beta\right)}^{2}\times S^{2}}{\begin{aligned} d^{2} \\ \\ Z\alpha=1.96 \\ Z\beta=0.842 \\ S= 36.16 \\ d=20 \end{aligned}} n^{'}=\frac{n}{\begin{aligned} 1-R \\ \\ n= 53 \\ d=0.10 \\ n^{'}=58 \end{aligned}}$ |
| --- |
| n = initial sample size to be calculated; Zα =1.96 (95% confidence level); Zβ = 0.842 (to detect a mean difference of 20 points); S = Variance of the quantitative variable that the control or reference group has; $d$ = Minimum value of the difference to be detected (quantitative data); q=1−p; e=accepted margin of error; n′: definitive sample size; R: expected proportion of losses expressed as a fraction |

**Supplementary Figure 1.** Sample size formula for Physical scale of the 36-Item Short Form Health Survey Questionnaire (SF-36)

| $n=\frac{{2\left( Z\alpha+ Z\beta\right)}^{2}\times S^{2}}{\begin{aligned} d^{2} \\ \\ Z\alpha=1.96 \\ Z\beta=0.842 \\ S= 29.99 \\ d=20 \end{aligned}} n^{'}=\frac{n}{\begin{aligned} 1-R \\ \\ n= 35 \\ d=0.10 \\ n^{'}=38 \end{aligned}}$ |
| --- |
| n = initial sample size to be calculated; Zα =1.96 (95% confidence level); Zβ = 0.842 (to detect a mean difference of 20 points); S = Variance of the quantitative variable that the control or reference group has; $d$ = Minimum value of the difference to be detected (quantitative data); q=1−p; e=accepted margin of error; n′: definitive sample size; R: expected proportion of losses expressed as a fraction |

**Supplementary Figure 2.** Sample size formula for Mental scale of the 36-Item Short Form Health Survey Questionnaire (SF-36)
